# Enzyme Immobilization by Inkjet Printing on Reagentless Biosensors for Electrochemical Phosphate Detection

**DOI:** 10.3390/bios14040168

**Published:** 2024-03-30

**Authors:** Dongxing Zhang, Yang Bai, Haoran Niu, Lingyun Chen, Junfeng Xiao, Qiuquan Guo, Peipei Jia

**Affiliations:** 1 Shenzhen Institute for Advanced Study, University of Electronic Science and Technology of China, Yesun Industry Zone, Guanlan Street, Shenzhen 518110, China; zhangdongxing@uestc.edu.cn (D.Z.); 202211040929@std.uestc.edu.cn (H.N.); 202222280936@std.uestc.edu.cn (L.C.); jxiao47@uwo.ca (J.X.); 2 Department of Biomedical Engineering, Western University, 1151 Richmond Street, London, ON N6A 3K7, Canada; ybai223@uwo.ca

**Keywords:** enzyme immobilization, inkjet printing, screen-printed electrode, reagentless biosensor, phosphate detection

## Abstract

Enzyme-based biosensors commonly utilize the drop-casting method for their surface modification. However, the drawbacks of this technique, such as low reproducibility, coffee ring effects, and challenges in mass production, hinder its application. To overcome these limitations, we propose a novel surface functionalization strategy of enzyme crosslinking via inkjet printing for reagentless enzyme-based biosensors. This method includes printing three functional layers onto a screen-printed electrode: the enzyme layer, crosslinking layer, and protective layer. Nanomaterials and substrates are preloaded together during our inkjet printing. Inkjet-printed electrodes feature a uniform enzyme deposition, ensuring high reproducibility and superior electrochemical performance compared to traditional drop-casted ones. The resultant biosensors display high sensitivity, as well as a broad linear response in the physiological range of the serum phosphate. This enzyme crosslinking method has the potential to extend into various enzyme-based biosensors through altering functional layer components.

## 1. Introduction

Electrochemical biosensing has become increasingly prominent in point-of-care research [1,2]. Among the various types of electrochemical biosensors, enzyme-based variants utilizing screen-printed electrodes have attracted significant attention from researchers due to their high specificity, portability, low cost, and potential for mass production through miniaturization [3]. Enzymes, serving as the sensing unit, need to possess high sensitivity toward the target analyte, as well as long-time stability and strong binding to the receptor/working electrode [4,5]. Therefore, employing effective enzyme immobilization strategies is crucial in enhancing a biosensor’s performance [6]. Common enzyme immobilization methods include gel entrapment, physical adsorption, and cross-linking [7]. While the first two approaches are often favored for their simplicity, they are plagued by limitations such as significant diffusion barriers, potential enzyme leakage, and weak bonding to the electrode surface, which compromise the stability and reproducibility of the biosensors. To address these challenges, crosslinking methods have been developed for enzyme immobilization which aim to establish robust binding. Typically, these methods involve the use of glutaraldehyde or other bifunctional agents, occasionally in combination with a protein, such as bovine serum albumin [8]. The cross-linking process is commonly executed through the drop-casting technique due to its operational simplicity and rapid reaction [9]. However, the low reproducibility of drop-casted surfaces, the coffee ring effect, and the low productivity have collectively hindered the application of drop-casted biosensors [10].

The inkjet printing technique has emerged as a prominent fabrication approach for biosensors as it provides advantages such as contactless printing, optimal material usage, and customization capabilities. Inkjet printing has been successfully employed in the fabrication of various biosensors, including glucose sensors [11,12], hydrogen peroxide sensors [13], ascorbic acid sensors [14], lysozyme sensors [15], and cholesterol sensors [12]. As a drop-on-demand manufacturing technology, inkjet printing enables more uniform deposition and higher production compared to the drop-casting technique. However, addressing printhead clogging due to the high viscosity of crosslinked glutaraldehyde and enzyme gel solutions remains a challenge. Researchers have made modifications to inkjet printing processes to mitigate this issue. For example, enzymes can be immobilized by exposing them to the vapor of glutaraldehyde. In these cases, the immobilized enzymes retain their activity after the printing and crosslinking. Nevertheless, this hybrid method adds complexity to the fabrication process. Additionally, reliance on an external electrochemical cell remains a concern for on-site biosensor use.

Inspired by a preloading strategy [16], substrates required for enzyme-catalyzed reactions can be preloaded onto the screen-printed electrodes using inkjet printing. This strategy eliminates the need for additional reagents during the sensing process [17,18], offering flexible fabrication methods for biosensors by precisely positioning a small amount of materials, and takes advantage of controlling the unnecessary wastage of enzymes and potential contamination [19]. In recent years, a number of enzyme-based glucose sensors have been developed for diabetes diagnosis [11,20]. Bihar E, et al. [21] used inkjet-printing technology for the rapid and low-cost deposition of all the components of this glucose sensor, from the electronics to the biorecognition elements, on commercially available paper substrates. Demuru S, et al. [22] demonstrated fully inkjet-printed graphene-gated organic electrochemical transistors (OECTs) on polymeric foil for the enzymatic-based biosensing of glucose. It is anticipated that inkjet printing will become a routine method for developing various enzyme-based biosensors, offering enhanced efficiency and versatility in their fabrication.

In this study, we devised a surface functionalization method utilizing inkjet printing to crosslink enzymes, thereby enabling electrochemical phosphate detection with reagentless biosensors. Our approach involved immobilizing enzymes through inkjet printing, incorporating both the enzyme layer and the crosslinking layer. This eliminated the need for the previous two-step cross-linking procedure, which required printing enzymes followed through exposure to glutaraldehyde vapor. Our innovative approach preloaded the necessary reagents onto the sensing area, eliminating the requirement for an external electrochemical cell. For our mono-enzyme phosphate sensors, we chose pyruvate oxidase as the model enzyme. An accurate measurement of phosphate levels in serum is crucial for clinical diagnosis, particularly for conditions such as hyperphosphatemia, cardiovascular diseases, and chronic kidney disease. The enzyme layer was comprised of pyruvate oxidase and cofactors, while the subsequent printing of glutaraldehyde formed a crosslinking layer anchoring the enzyme layer onto the sensing electrode. We optimized sensor performance by exploring the printing parameters, ink components, buffer pH, and test solution pH. The resulting biosensors exhibited high sensitivity and a broad linear response within the physiological range of the serum phosphate. Our innovative strategy of inkjet printing for surface functionalization enabled efficient enzyme crosslinking, thereby enhancing the reproducibility and effectiveness of enzyme-based biosensors. This approach holds promise for advancing the field of biosensing technology.

## 2. Materials and Methods

### 2.1. Chemicals and Instruments

Pyruvate oxidase from *microorganism* (PyOD, E.C.1.2.3.3, 10 U/mg) was purchased from Toyobo (New York, NY, USA). Multiwall carbon nanotubes (5–15 µm), which were functionalized before use, were purchased from Shenzhen Nanotech Port Co., Ltd. (Shenzhen, China). Bovine serum albumin (BSA), Triton X-100, hydrochloric acid, nitric acid, sulfuric acid, potassium ferricyanide (III) (K_3_Fe(CN)_6_), glutaraldehyde (GLA), Nafion^®^ (5 wt.%), magnesium sulfate, sodium bicarbonate, citric acid, and sodium citrate dihydrate were all obtained from Sigma-Aldrich. Thiamine pyrophosphate (TPP), flavin adenine dinucleotide (FAD), magnesium chloride (MgCl_2_), and pyruvic acid were purchased from TCI. All reagents were used as received unless otherwise stated.

All solutions were prepared in deionized water (18.2 MΩ·cm^−1^). A 50 mM phosphate stock solution (pH 6.0) and a 25 mM citrate buffer (CB, pH 5.6) were prepared according to normal protocol and stored at 4 °C. A basic artificial serum solution for sensor validity studies was made by mixing 0.68 g sodium chloride, 0.02 g calcium chloride, 0.04 g potassium chloride, 0.01 g magnesium sulfate, and 0.22 g sodium bicarbonate together in 0.1 L DI water and was then stored at 4 °C [23]. Then, different concentrations of phosphate solution were dissolved in the basic artificial serum solution.

Screen printed electrodes (C10) were purchased from Mxense Bio-Tech Co., Ltd. (Shanghai, China) with a three-electrode system: a working electrode (material: carbon, geometry area: 0.053 cm^2^), a counter electrode (material: carbon), and a reference electrode (material: Ag/AgCl). SNB-1 Viscosimeter (Karoth Shanghai, China) was used to measure the viscosity at room temperature. BZY101 Automatic Surface Tensiometer (Vetus, Hefei, Anhui, China) was used to characterize the surface tension. A Fujifilm DIMATIX Materials Printer DMP-2831 (FUJIFILM Dimatix, Inc., Santa Clara, CA, USA) was used to carry out printing experiments. UV-vis absorption spectra were recorded with a Cary 100 UV-visible spectrophotometer (Agilent, Santa Clara, CA, USA). A Nicolet 6700 spectrophotometer (Thermo Nicolet) was used to record the Fourier transform infrared (FTIR) spectra under transmittance mode. Scanning electron microscopy (SEM, Hitachi S-4500) was used to observe the morphology at a 10 kV accelerating voltage. Atomic force microscope (AFM, Dimension V AFM) was performed to examine the surface roughness of the working electrode. Cyclic voltammetry (CV) measurements were conducted with a CHI potentiostat (1200C, Shanghai Chen Hua Instrument Co., Ltd., Shanghai, China).

### 2.2. Functionalization of the MWCNTs

To improve the solubility of MWCNTs in the water-based ink, as well as enhance their affinity with enzymes, MWCNTs were functionalized with -COOH groups (see Figure S1) through a modification of the oxidation method reported by Yu-Chun Chiang et.al. [24,25]. First, MWCNTs were soaked in hydrochloric acid for 24 h. Then, the MWCNTs were precipitated from the solution by centrifuging for 15 min and then rinsing with deionized water three times. Next, MWCNTs were dried in the air, and 100 mg of MWCNTs were added in 40 mL nitric acid and sulfuric acid solution with a weight ratio of 1:3. The obtained mixture was heated and stirred at 50 °C for 24 h. Following this, 100 mL of distilled water was added, and the mixture underwent centrifugation. The process of washing with distilled water continued until the dispersion reached neutrality. The dispersion was then dried in a vacuum at 50 °C overnight. Finally, the obtained functionalized MWCNTs were stored in a dry state at room temperature.

### 2.3. Ink Formulation

To prepare the printing ink of the enzyme layer (enzyme ink), we prepared a solution containing 16 U/mL pyruvate oxidase, 25 µM TPP, 6 µM FAD, 2 mM MgCl_2_, 2 mM pyruvic acid, 0.5 mg/mL functionalized MWCNTs (filtered by 0.2 µm filter before use), 2.4% *w*/*v* BSA, and 0.0075% *v*/*v* Triton X-100 using the prepared stock solutions. The enzyme ink was gently mixed and left for 30 min before use. For the ink of the crosslinking layer (GLA ink), 2.5% *w*/*v* GLA and 0.006% *v*/*v* Triton X-100 was prepared in deionized water. The ink for the top protective layer (Nafion ink) consisted of 1.5% *w*/*v* Nafion and 0.005% *v*/*v* Triton X-100.

Different concentrations of Triton×-100 were used to modulate ink surface tension according to the printing performance and droplet surface wetting. Viscosities were also optimized within the range of ink printability suggested by the printer manufacturer.

### 2.4. Settings of Printing Parameters

**Figure 1:**
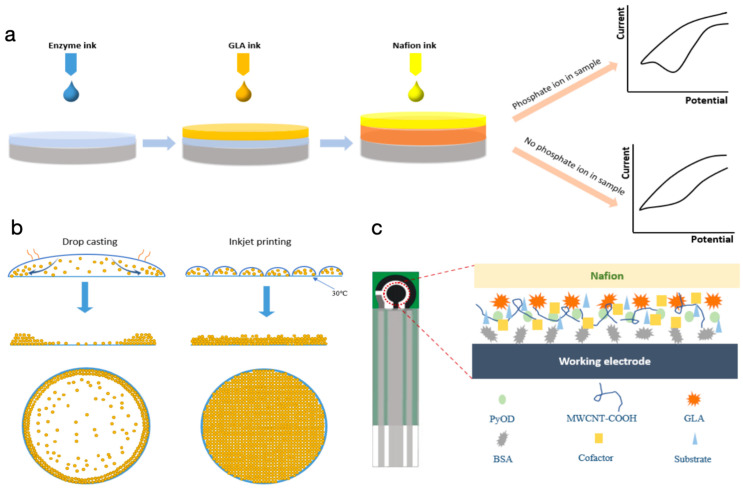
Enzyme immobilization (crosslinking) by inkjet printing on reagentless biosensors. (**a**) The surface functionalization process on the working electrode and the sensor sensing mechanism. (**b**) The drying process of the droplet on the electrode, side view, and top view of the particle distribution status by drop casting method and the inkjet printing method. (**c**) Schematic diagram for the cross section of the functionalized working electrode.

The printing waveforms (see Figure S2) and printing parameters were optimized based on our previous studies to ensure stable printing while maintaining high enzyme activity [18]. A fiducial camera was used to ensure the uniform deposition of the pattern through observing the relative position of adjacent drops during the printing process [26]. The drop spacing was set as 20 µm. The screen-printed electrode was heated to 30 °C while printing for the quick evaporation of the printed ink. Droplet counts of different layers were programmed through Pattern Editor Module to ensure defined amounts of material deposition. The schematic diagram for the cross section of the functionalized working electrode is shown in Figure 1. Specifically, 20 layers of enzyme ink (459,330 counts) were first printed on the working electrode, followed by 10 layers of GLA ink (229,665 counts). The first two layers reacted and formed a crosslinking composite. In the end, 10 layers of Nafion ink (229,665 counts) were printed on top of it to form a protective and selective membrane.

### 2.5. Voltammetric Experiments Using the Proposed Biosensor

30 µL of a prepared phosphate solution or artificial serum solution was dropped onto the sensing area of the biosensor. The potential range was set between 0 and 0.6 V at a scan rate of 50 mV/s. All electrochemical responses were measured three times to obtain the average. All experiments were performed based on these above settings unless otherwise stated.

## 3. Results and Discussion

### 3.1. Mechanism for the Biosensor through the Proposed Printing Strategy

Figure 1a shows the surface functionalization process of the working electrode and the working mechanism of the proposed phosphate sensor. The bottom layer (depicted in blue) represents the MWCNTs-COOH/enzyme/cofactor/substrate/BSA composite. The introduction of cofactors into this layer streamlined the analyte reaction, simplifying the traditionally intricate reagent addition process in enzyme-based electrochemical biosensor fabrication. MWCNTs modified with carboxyl groups were used to improve the affinity to the enzyme protein, and to increase the surface area to produce more enzyme loading and superior electrochemical property [25,27]. BSA was also integrated into the enzyme layer as the stabilizing and sacrificial agent in the printing and crosslinking process [8,28,29]. Throughout the printing process, the screen-printed electrode was heated to facilitate droplet evaporation and minimize liquid spreading, ensuring higher deposition precision and uniformity on the working electrode. Subsequently, GLA (orange layer) was printed to form a crosslinking composite (shown in red) together with the bottom layer. This interaction occurred through the reaction with the amino groups of lysine distributed on the external surface of BSA/enzyme with the GLA [28]. A semipermeable nafion layer (yellow layer) was then printed as a protective and ion selective membrane. The biosensor can work to detect the analyte by measuring the oxidation current of the reaction product H_2_O_2_ which is produced because of the following reactions:

pyruvate + phosphate + O_2_ → acetyl phosphate + H_2_O_2_ + CO_2_ (1)

H_2_O_2_ → 2H^+^ +2e^−^ +O_2_ (2)

A cathodic peak (oxidation of the hydrogen peroxide) can be observed upon sweeping of the potential on the working electrode.

### 3.2. Comparison of Surface Functionalization Performance between Drop-Casting and Inkjet-Printing Methods

**Figure 2:**
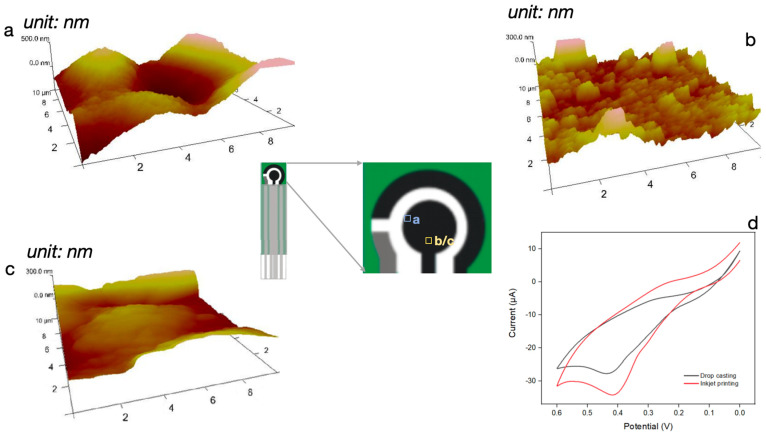
(**a**) AFM image of the “coffee ring” effect by drop casting method. (**b**) AFM image of the area between the center and the “coffee ring” on the working electrode by drop casting method. (**c**) AFM image of the same area as in (**b**) but for the inkjet printing method. (**d**) CV curves of the functionalized electrode fabricated by drop casting method and inkjet printing method in the presence 2 mM phosphate. Scan rate: 50 mV/s.

AFM was used to demonstrate the mitigation of the “coffee ring” effect by comparing the surface roughness, as seen in Figure 2. The inkjet printing method has proven highly effective in eliminating the “coffee ring” effect that typically occurs during the use of the traditional drop casting method [30]. In drop casting, when ink is applied onto the working electrode, the ingredients of the ink would be pushed to the edge of the drop due to the capillary flow during evaporation, causing a “coffee ring” on the edge (Figure 1b). The formed “coffee ring”, shown in Figure 2a, has an obvious height difference between the outer and central area. The middle part of the drop-casted electrode showed a bumpy morphology that indicated less particle distribution (Figure 2b). In contrast, micro-droplets deposited by inkjet printing can evaporate individually on the electrode, allowing for a more uniform distribution of functional particles (Figure 1b); thus, the coffee ring effect was largely mitigated (Figure 2c). This mitigation effect can be interpreted as resulting from the smaller droplet size (decrease the coffee ring size [31]), substrate heating procedure (accelerate evaporation time [26]), and the inclusion of surfactant (which increases Magaroni flow [32]).

CV responses of the functionalized electrode by drop casting and inkjet printing were plotted in Figure 2d to compare their effects on analytical performance. A higher and more evident oxidation peak was shown for the inkjet printing method. The effective surface area of the electrode can be obtained by the Randles-Servick equation [33,34]:

*I*_p_ = 268,600*n*^3/2^*AD*^1/2^*Cν*^1/2^ (3)

where *I*_p_ is the peak current, *n* is the electron transfer number, *A* is the electroactive surface area, *D* is the diffusion coefficient of the electrolyte, *C* is the electrolyte concentration, and *ν* is the scan rate. Keeping other parameters constant, *I*_p_ is proportional to the effective surface area *A*. The effective surface area of inkjet-printed modified electrode was calculated to be around 1.5 times higher than the drop casted electrode, which helps explain the superiority for electrochemical measurement. Moreover, machine-controlled inkjet printing offers advantages for scale-up production compared to drop casting, as it enables the fabrication of a more uniformly functionalized working electrode [10].

### 3.3. Optimization of the Experimental Conditions

**Figure 3:**
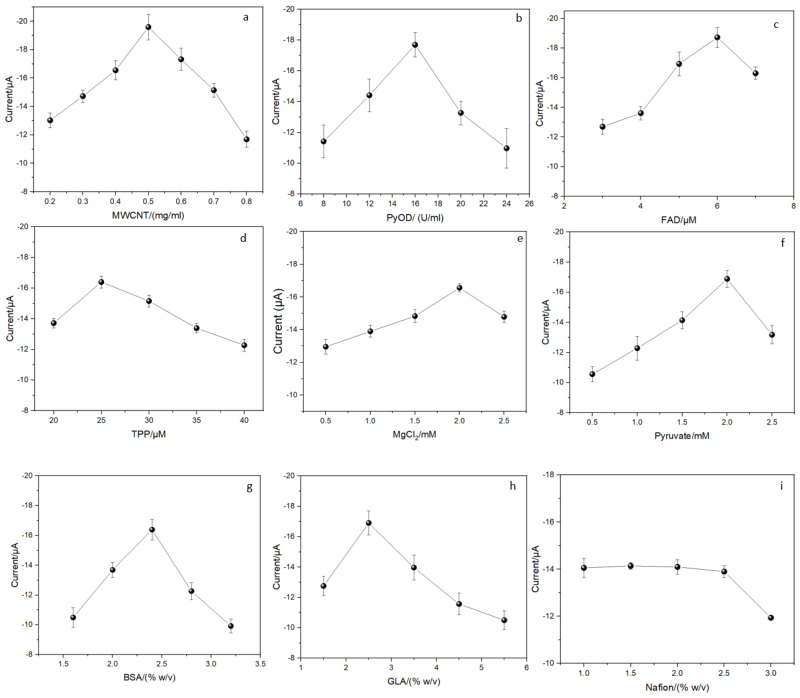
Effects of the concentration of (**a**) MWCNTs-COOH, (**b**) PyOD, (**c**) FAD, (**d**) TPP, (**e**) MgCl_2_, (**f**) pyruvate, (**g**) BSA, (**h**) GLA, (**i**) Nafion on the oxidation peak current of the cyclic voltammogram obtained from the modified electrode. Phosphate concentration measured was 2 mM.

The impact of using functionalized MWCNTs-COOH on the electrochemical performance of the biosensor was also investigated, and the improvements are shown in Figure 3a. Notably, we conducted thorough measurements on the absence of phosphate ions to establish a baseline, revealing a negligible background current magnitude. For the sake of simplicity in narration, the following analysis concerning the influence of variables on the electrochemical performance was indeed conducted by utilizing the measured data after subtracting the negligible background current in our manuscript. It was found that functionalized MWCNTs play a pivotal role as electron transfer shuttles, significantly influencing the electrochemical reaction. The concentration of functionalized MWCNTs was systematically varied from 0.2 mg/mL to 0.8 mg/mL. The highest peak current was observed at a concentration of 0.5 mg/mL. Lower concentrations of MWCNTs (≤0.5 mg/mL) exhibited a positive increase in current response due to enhanced MWCNTs-enzyme binding. Conversely, higher concentrations of MWCNTs (≥0.5 mg/mL) resulted in background interference and a saturation of the ink composite, thereby impeding the reaction [35,36]. Thus, functionalized MWCNTs at 0.5 mg/mL was selected for all subsequent experiments in this study.

In Figure 3b, the effect of pyruvate oxidase on the oxidation current is depicted. Enzymes concentrations ranging from 8 U/mL to 24 U/mL were employed in the measurements. The peak current increased with the enzyme concentration until reaching the highest value at 16 U/mL. This phenomenon can be attributed to the fact that low enzyme loading leads to a slower rate of enzyme catalysis and, consequently, a lower current, while higher enzyme concentrations create a higher diffusion barrier that restricts the current response [37].

Optimization for the concentration of cofactors (FAD, TPP, Mg^2+^) was conducted by assessing enzyme loading, as illustrated in Figure 3c–e. These cofactors exhibited similar trends of increasing and decreasing peak currents with varying concentrations. Since cofactors bind with the enzyme around the active sites, the current will decrease as the concentrations of cofactors are too high to hinder the enzyme catalytic reaction. The maximum peak currents for FAD, TPP and Mg^2+^ were found at the concentration of 6 µM, 25 µM and 2 mM respectively. All subsequent experiments were based on these values.

The effect of the concentration of the substrate (pyruvate) was also investigated, with results shown in Figure 3f. The peak current reached its highest value at 2 mM and then declined due to saturation. Therefore, the optimal pyruvate concentration of 2 mM was selected for all subsequent experiments.

The optimization of crosslinking agents concentrations (BSA and GLA) is shown in Figure 3g,h. BSA serves as an inert lysine-rich auxiliary protein and protective spacer crucial for effective enzyme crosslinking process [37,38]. The concentration of BSA defines the spacing of enzyme molecules in the crosslinking composite. Excessive BSA concentration leads to a decrease in response current due to high protein content [39]. GLA plays an important role in crosslinking the components of the bottom layer to form a gel composite. Thus, deficient GLA result in peak current reduction due to insufficient crosslinking, while excessive GLA can occupy the active sites of enzyme which hinders the reaction [40]. The optimal concentrations of BSA and GLA were determined to be 2.4% *w*/*v* and 2.5% *w*/*v*, respectively. We also investigated the optimal concentration of Nafion. The peak current for nafion presented a minimal change at the low concentration range of 1.0% *w*/*v* to 2.5% *w*/*v* and began to drop after 3% *w*/*v* because of increased diffusion barrier (Figure 3i). A concentration of 1.5% *w*/*v* was chosen and used in all subsequent experiments due to a relative higher current response.

**Figure 4:**
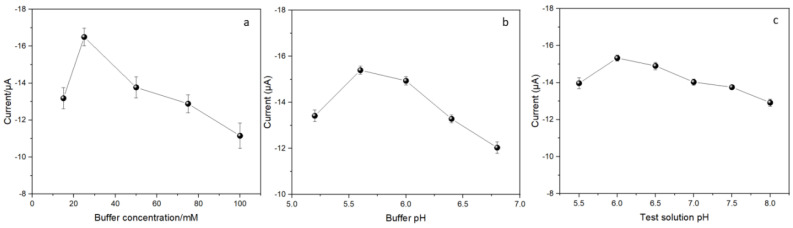
Effects of the (**a**) the concentration of the citrate buffer, (**b**) the pH of the citrate buffer, (**c**) the pH of the test solution on the oxidation peak current of the cyclic voltammogram obtained from the modified electrode. Phosphate concentration measured was 2 mM.

The concentration and pH of the citrate buffer for the enzyme ink were scrutinized, as shown in Figure 4a,b. The buffer concentration ranged from 15 mM to 100 mM. The optimal concentration of citrate buffer was determined to be 25 mM, with higher buffer concentrations resulting in decreased produced current. This phenomenon can be attributed to the fact that larger buffer concentrations possess greater buffering capacity but may impede the movement of H_2_O_2_, as well as electron transfer to the electrode [37,41]. Furthermore, the pH of the citrate buffer was explored across a range from 5.2 to 6.8, with pH 5.6 yielding the highest peak current. Deviations from this optimal pH level, whether lower or higher, led to a decrease in current due to reduced enzyme activity and the potential enzyme denaturation [37,42].

Therefore, the concentration and pH of the citrate buffer were standardized at 25 mM and 5.6, respectively, for all subsequent experiments. The pH of the test solution was also optimized by comparing the peak current obtained from pH 5.5 to pH 8. As shown in Figure 4c, only a relatively small variation (<6.7%) of the peak current happened within the pH range of 6 to 7, indicating its potential suitability for detecting phosphate levels in body fluids.

### 3.4. Evaluation of the Functionalized Working Electrode

**Figure 5:**
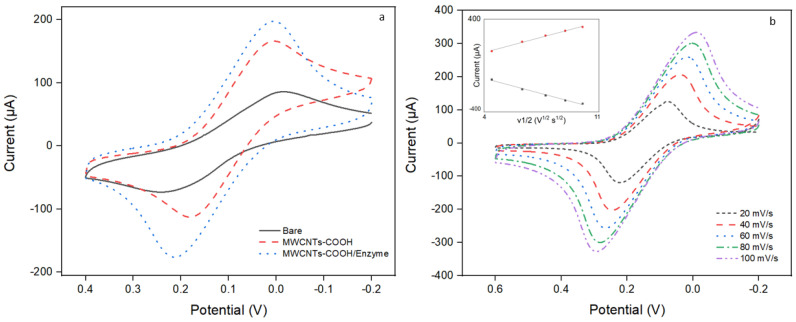
Electrochemical performance of the modified electrode. (**a**) CV curves of bare electrode (solid line), MWCNTs-COOH/substrate/cofactor electrode (dashed line), MWCNTs-COOH/enzyme/substrate/cofactor electrode (dot line) in 0.1 M KCl which contained 10 mM K_3_Fe(CN)_6_. Scan rate: 50 mV/s. (**b**) CV curves of the MWCNTs-COOH/enzyme/substrate/cofactor electrode in 0.1 M KCl which contained 10 mM K_3_Fe(CN)_6_ at scan rates of 20, 40, 60, 80 and 100 mVs^−1^. Inset is the plot of peak current vs. square root of scan rates.

CV responses of different electrodes to the K_3_Fe(CN)_6_ solution (Figure 5a) showed that the addition of MWCNTs facilitated the electron transfer process. The presence of MWCNTs-COOH (dashed line) resulted in decreased peak-peak separation and increased peak current (approximately three times higher than that of the bare electrode) which suggested the evident improvement for the electron transfer kinetics [43]. The combination of MWCNTs-COOH and enzyme electrode (dot line) showed even higher peak current than MWCNTs-COOH electrode, which resulted from the syngeneic effect of MWCNTs-COOH and enzyme for electron transfer. In Figure 5b, both the anodic/cathodic peak current, as well as peak-peak separation, increased with the scan rate from 20 to 100 mV/s. This suggested that the electrode reaction was a surface-confined and diffusional-controlled process [37]. This can be explained by the fact that certain enzymes are capable of direct electron transfer between their active-site cofactor and an electrode, while others rely on cytochrome domains or subunits as intrinsic redox mediators. These cytochromes serve the physiological function of transferring electrons between the active-site cofactor and a redox partner protein [44]. Therefore, the enhanced response observed in the presence of the enzyme likely stems from the facilitation of electron transfer processes, highlighting the complex interplay between electrode composition and enzymatic activity in electrochemical systems. Additionally, a linear relationship of the peak current and the square root dependence of the scan rate was acquired (seen in the inset of Figure 5b), which indicates that the electron transfer process was quasi-reversible.

### 3.5. Evaluation of the Constructed Biosensor

**Figure 6:**
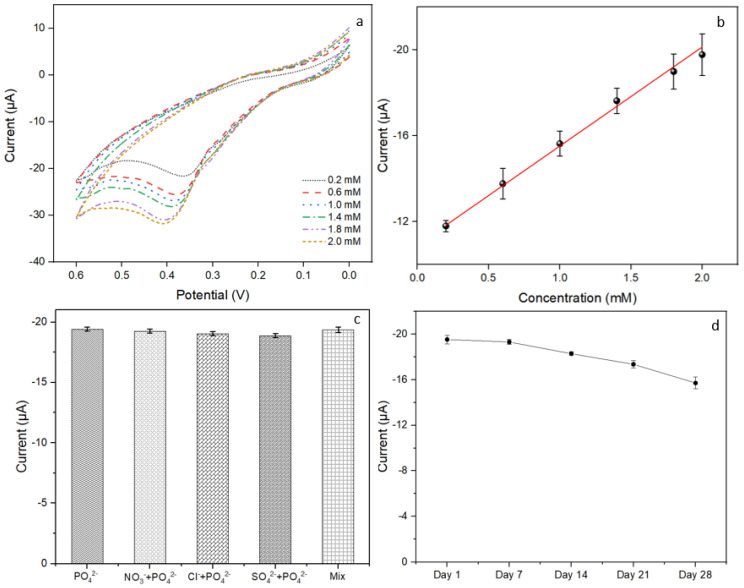
Current responses of the sensor to different concentration of phosphate by cyclic voltammetry. (**a**) Concentration increased with the direction of the orange arrow (0.2 to 2.0 mM). (**b**) Calibration curve for different concentration of phosphate. (**c**) Interference test. (**d**) Sensor stability test.

Figure 6a shows a proportional increase in peak current with phosphate concentration. The peak current of modified anodic electrode appeared at around the potential of 0.4 V, in agreement with the literature [45]. We achieved a wide linear range of 0.2 mM to 2.0 mM (R^2^ = 0.997), with a detection limit of 0.13 mM (refer to Figure 6b). This range encompasses the clinically relevant concentration range, as the normal serum phosphate concentration in adults typically ranges from 0.81 to 1.45 mM [46]. Notably, an intercept occurs in Figure 6b because the peak current, ip, of the reversible redox process is described by the Randles-Sevcik equation. Our fabricated biosensor presented superiorities in terms of electrode composition, sensor linear range, use of reagents, and manufacturing process; Table 1 compares different electrochemical sensors made by other fabrication methods. Though the sensor fabricated by photolithography method [47] has shown an extremely wide linear range, the inkjet printing is much more cost-effective and simple for manufacturing. Compared with the electrode modification by drop-casting technology [48] for phosphate detection, our inkjet printing method demonstrates high potential for future mass production. In addition, the substrate preloading strategy in our study enables reagentless measurement using SPE sensors, surpassing other reagent treatment methods that require SPE sensing platform [40,49,50]. Compared with the multi-enzyme biosensor for phosphate detection [51], the mono-enzyme system that we proposed is a more stable and cost-effective analytical system [52]. The use of SPE in our sensor is evidently superior to the traditional electrochemical cell sensing platform that can be further utilized in the point-of-care testing [51,53,54]. Also, our fabricated biosensor has a wider linear range compared with the ranges achieved by AuNWs [37] (12.5–1000 µM) and Pt/Au alloy [38] (0.248–1.456 mM) modified electrodes. The improvement is attributed to the uniform modification of the electrode surface achieved by the inkjet printing method, with the inclusion of functionalized MWCNTs facilitating rapid electron transfer during reaction.

The assessment of the interference effects from various anions is a standard practice in biosensor research due to the necessity of ensuring selectivity and reliability in sensing applications. Selectivity tests were conducted using phosphate solutions both with/without the addition of commonly-used interference species (NO_3_^−^, Cl^−^, SO_4_^2−^ and their mixture), as depicted in Figure 6c. Results revealed a negligible effect (<2%) from all the interferents on the sensing response to the phosphate. These findings indicated the proposed sensor had a satisfactory selectivity. Stability testing was explored and shown in Figure 6d. A batch of optimized sensors were vacuum sealed and stored at 4 °C before use. The results showed that the sensor retained 89% of the initial response after three weeks, indicating a high stability of the prepared biosensor.

Reproducibility of the proposed biosensor was also assessed by preparing five biosensors under the optimized condition. The peak current response to 2 mM phosphate showed a relative standard deviation of less than 4%, affirming the reproducibility of the fabrication.

Furthermore, three artificial serum samples of different concentration were prepared and measured to validate the applicability of the proposed sensor (refer to Table 2). The recovery of biosensor ranged from 98.9% to 103% suggesting its potential for the serum phosphate detection. Additionally, the cost for the proposed electrochemical biosensor is less than 1.34 USD (refer to Table S1) indicating the feasibility of mass fabrication.

## 4. Conclusions

This study introduces a straightforward and highly effective surface functionalization strategy, utilizing inkjet printing, to achieve enzyme crosslinking for the construction of a reagentless enzyme-based biosensor. This innovative method eliminates the need for additional post-treatment, thus streamlining the process to only involve printing procedures. Consequently, it enables a more uniform deposition and enhances analytical performance as compared to biosensors produced by traditional drop-casting methods. The formulation of ink and printing routines for multiple functionalization layers in sensor fabrication were meticulously presented, providing valuable insights for the development of inkjet-printed biosensors. In addition, the efficacy of this strategy was validated through successful phosphate detection in artificial serum samples. Combining this surface functionalization approach with the substrate preloading strategy, the constructed biosensor exhibits potential for industrialization in on-site analyte detection with minimal sample requirements (30 µL). Notably, as a simple, affordable, labor-free, and reagentless approach, this sensor fabrication approach can be readily adapted to the construction of various enzyme-base biosensors by simply modifying the enzyme in the ink components.

## Figures and Tables

**Table 1:** Comparison of electrochemical sensors reported in literature for phosphate detection.

Electrode	Composition	Linear Range	Reagents Addition	Manufacturing Method	Ref
Co microelectrodes	e-beam evaporator of Au and Cobalt	0.01–10 mM	Potassium hydrogen phthalate	Photolithography	[47]
GLA/PyOD/CoPC/SPE	PyOD cross-linked with GLA	2.5–130 µM	MOPS, pyruvic acid, MgSO_4_, NaCl	Drop cast	[40]
CB/SPE	CB	0.5–100 µM	Molybdate, H_2_SO_4_, KCL	Drop cast	[49]
CB/SPE	CB and reaction substrate	10–300 µM	None	Drop cast	[48]
PyOD/SPE	Gel-entrapment of PyOD	7.5–625 µM	Pyruvic acid, FAD, TPP, MgCl_2_, citrate buffer	Drop cast	[50]
PyOD/MWCNT/GCE	Gel-entrapment of PyOD and MWCNT	1–100 µM	Sodium pyruvate, TPP, FAD, Mg^2+^	Drop cast	[53]
Polyelectrolyte/ PyOD/CE	Physical adsorption of polyelectrolyte and PyOD on the carbon electrode	0.05–1.25 mM	Pyruvate and HEPES buffer	Drop cast	[54]
Multienzyme/ carbon paste	Three kinds of enzymes and carbon paste electrode	2–250 mM	NAD^+^, MgCl_2_, Os(l,10-phenanthroline-5,6-dione)2Cl_2_), Tris buffer	Drop cast	[51]
AuNWs/cofactor/BSA/PyOD/GLA	Crosslinking of cofactors and PyOD by BSA and GLA	12.5–1000 µM	Pyruvic acid, MgCl_2_, citrate buffer	Drop cast	[37]
Pt/Au/cofactor/BSA/PyOD/GLA	Crosslinking of cofactors and PyOD by BSA and GLA	0.248–1.456 mM	Sodium pyruvate, MgCl_2_, NaOH, citrate buffer	Electrodeposition Drop cast	[38]
MWCNT-COOH/ cofactor/substrate/BSA/PyOD/GLA/SPE	Crosslinking of MWCNTs, cofactors, substrate and PyOD by BSA and GLA	0.2–2 mM	None	Inkjet printing	This work

**Table 2:** Detection of phosphate in the artificial serum (n = 3).

Phosphate Concentration in Sample/mM	Founded Phosphate/mM	Recovery/%	RSD/%
1.50	1.49	99.1	1.7
1.25	1.24	98.9	3.3
1.00	1.03	103	6.4

## Data Availability

Data are contained within the article and Supplementary Materials.

## Supplementary Materials

The following supporting information can be downloaded at: https://www.mdpi.com/article/10.3390/bios14040168/s1. See Refs. [55,56,57].

## References

[1] Wang M., Yang Y., Min J., Song Y., Tu J., Mukasa D., Ye C., Xu C., Heflin N., McCune J.S. (2022). A wearable electrochemical biosensor for the monitoring of metabolites and nutrients. Nat. Biomed. Eng..

[2] Bariya M., Nyein H.Y.Y., Javey A. (2018). Wearable sweat sensors. Nat. Electron..

[3] Bollella P. (2022). Enzyme-based amperometric biosensors: 60 years later… Quo Vadis?. Anal. Chim. Acta.

[4] Walter P., Podsiadły B., Wałpuski B., Jakubowska M. (2018). Common configurations and challenges in screen-printed enzymatic electrochemical biosensors. Proceedings of the Photonics Applications in Astronomy, Communications, Industry, and High-Energy Physics Experiments.

[5] Lakhera P., Chaudhary V., Jha A., Singh R., Kush P., Kumar P. (2022). Recent developments and fabrication of the different electrochemical biosensors based on modified screen printed and glassy carbon electrodes for the early diagnosis of diverse breast cancer biomarkers. Mater. Today Chem..

[6] Chen H., Simoska O., Lim K., Grattieri M., Yuan M., Dong F., Lee Y.S., Beaver K., Weliwatte S., Gaffney E.M. (2020). Fundamentals, applications, and future directions of bioelectrocatalysis. Chem. Rev..

[7] Zhang R., Jiang J., Wu W. (2022). Scalably Nanomanufactured Atomically Thin Materials-Based Wearable Health Sensors. Small Struct..

[8] Sassolas A., Blum L.J., Leca-Bouvier B.D. (2012). Immobilization strategies to develop enzymatic biosensors. Biotechnol. Adv..

[9] Li H., Li A., Zhao Z., Li M., Song Y. (2020). Heterogeneous wettability surfaces: Principle, construction, and applications. Small Struct..

[10] Kaliyaraj Selva Kumar A., Zhang Y., Li D., Compton R.G. (2020). A mini-review: How reliable is the drop casting technique?. Electrochem. Commun..

[11] Zub K., Hoeppener S., Schubert U.S. (2022). Inkjet printing and 3D printing strategies for biosensing, analytical, and diagnostic applications. Adv. Mater..

[12] Wang X., Zhang M., Zhang L., Xu J., Xiao X., Zhang X. (2022). Inkjet-printed flexible sensors: From function materials, manufacture process, and applications perspective. Mater. Today Commun..

[13] Isailović J., Vidović K., Hočevar S.B. (2022). Simple electrochemical sensors for highly sensitive detection of gaseous hydrogen peroxide using polyacrylic-acid-based sensing membrane. Sens. Actuators B Chem..

[14] Pradela-Filho L.A., Gongoni J.L., Arantes I.V., De Farias D.M., Paixão T.R. (2023). Controlling the inkjet printing process for electrochemical (bio) sensors. Adv. Mater. Technol..

[15] Li Y., Liu Y., Bhuiyan S.R.A., Zhu Y., Yao S. (2022). Printed Strain Sensors for On-Skin Electronics. Small Struct..

[16] Cinti S., Moscone D., Arduini F. (2019). Preparation of paper-based devices for reagentless electrochemical (bio) sensor strips. Nat. Protoc..

[17] Bai Y., Guo Q., Xiao J., Zheng M., Zhang D., Yang J. (2021). An inkjet-printed smartphone-supported electrochemical biosensor system for reagentless point-of-care analyte detection. Sens. Actuators B Chem..

[18] Bai Y., Zhang D., Guo Q., Xiao J., Zheng M., Yang J. (2021). Study of the Enzyme Activity Change due to Inkjet Printing for Biosensor Fabrication. ACS Biomater. Sci. Eng..

[19] Derby B. (2008). Bioprinting: Inkjet printing proteins and hybrid cell-containing materials and structures. J. Mater. Chem..

[20] Aghababaie M., Foroushani E.S., Changani Z., Gounani Z., Mobarakeh M.S., Hadady H., Khedri M., Maleki R., Asadnia M., Razmjou A. (2023). Recent Advances In the development of enzymatic paper-based microfluidic biosensors. Biosens. Bioelectron..

[21] Bihar E., Wustoni S., Pappa A.M., Salama K.N., Baran D., Inal S. (2018). A fully inkjet-printed disposable glucose sensor on paper. npj Flex. Electron..

[22] Demuru S., Huang C.-H., Parvez K., Worsley R., Mattana G., Piro B., Noël V., Casiraghi C., Briand D. (2022). All-inkjet-printed graphene-gated organic electrochemical transistors on polymeric foil as highly sensitive enzymatic biosensors. ACS Appl. Nano Mater..

[23] Basiaga M., Paszenda Z., Walke W., Karasiński P., Marciniak J. (2014). Electrochemical Impedance Spectroscopy and corrosion resistance of SiO_2_ coated cpTi and Ti-6Al-7Nb alloy. Information Technologies in Biomedicine.

[24] Chiang Y.-C., Lin W.-H., Chang Y.-C. (2011). The influence of treatment duration on multi-walled carbon nanotubes functionalized by H_2_SO_4_/HNO_3_ oxidation. Appl. Surf. Sci..

[25] Noordadi M., Mehrnejad F., Sajedi R.H., Jafari M., Ranjbar B. (2018). The potential impact of carboxylic-functionalized multi-walled carbon nanotubes on trypsin: A Comprehensive spectroscopic and molecular dynamics simulation study. PLoS ONE.

[26] Soltman D., Subramanian V. (2008). Inkjet-Printed Line Morphologies and Temperature Control of the Coffee Ring Effect. Langmuir.

[27] Gupta S., Murthy C., Prabha C.R. (2018). Recent advances in carbon nanotube based electrochemical biosensors. Int. J. Biol. Macromol..

[28] Sheldon R.A., van Pelt S. (2013). Enzyme immobilisation in biocatalysis: Why, what and how. Chem. Soc. Rev..

[29] Du X., Durgan C.J., Matthews D.J., Motley J.R., Tan X., Pholsena K., Árnadóttir L., Castle J.R., Jacobs P.G., Cargill R.S. (2015). Fabrication of a flexible amperometric glucose sensor using additive processes. ECS J. Solid State Sci. Technol. JSS.

[30] Li H., Buesen D., Williams R., Henig J., Stapf S., Mukherjee K., Freier E., Lubitz W., Winkler M., Happe T. (2018). Preventing the coffee-ring effect and aggregate sedimentation by in situ gelation of monodisperse materials. Chem. Sci..

[31] Shen X., Ho C.-M., Wong T.-S. (2010). Minimal Size of Coffee Ring Structure. J. Phys. Chem. B.

[32] Mampallil D., Eral H.B. (2018). A review on suppression and utilization of the coffee-ring effect. Adv. Colloid Interface Sci..

[33] He B., Liu H. (2020). Electrochemical biosensor based on pyruvate oxidase immobilized AuNRs@ Cu_2_O-NDs as electroactive probes loaded poly (diallyldimethylammonium chloride) functionalized graphene for the detection of phosphate. Sens. Actuators B Chem..

[34] de Rooij M.R. (2003). Electrochemical methods: Fundamentals and applications. Anti Corros. Methods Mater..

[35] Yao L., Teng J., Zhu M., Zheng L., Zhong Y., Liu G., Xue F., Chen W. (2016). MWCNTs based high sensitive lateral flow strip biosensor for rapid determination of aqueous mercury ions. Biosens. Bioelectron..

[36] Guan W.-J., Li Y., Chen Y.-Q., Zhang X.-B., Hu G.-Q. (2005). Glucose biosensor based on multi-wall carbon nanotubes and screen printed carbon electrodes. Biosens. Bioelectron..

[37] Ogabiela E., Adeloju S.B., Cui J., Wu Y., Chen W. (2015). A novel ultrasensitive phosphate amperometric nanobiosensor based on the integration of pyruvate oxidase with highly ordered gold nanowires array. Biosens. Bioelectron..

[38] Cui J., Ogabiela E.E., Hui J., Wang Y., Zhang Y., Tong L., Zhang J., Adeloju S.B., Zhang X., Wu Y. (2015). Electrochemical Biosensor based on Pt/Au Alloy Nanowire Arrays for Phosphate Detection. J. Electrochem. Soc..

[39] Adeloju S.B., Lawal A.T. (2011). Fabrication of a bilayer potentiometric phosphate biosensor by cross-link immobilization with bovine serum albumin and glutaraldehyde. Anal. Chim. Acta.

[40] Gilbert L., Jenkins A.T.A., Browning S., Hart J.P. (2011). Development of an amperometric, screen-printed, single-enzyme phosphate ion biosensor and its application to the analysis of biomedical and environmental samples. Sens. Actuators B Chem..

[41] Lawal A.T., Adeloju S.B. (2013). Polypyrrole based amperometric and potentiometric phosphate biosensors: A comparative study B. Biosens. Bioelectron..

[42] Luo X.-L., Xu J.-J., Du Y., Chen H.-Y. (2004). A glucose biosensor based on chitosan–glucose oxidase–gold nanoparticles biocomposite formed by one-step electrodeposition. Anal. Biochem..

[43] Wang J., Musameh M. (2004). Carbon nanotube screen-printed electrochemical sensors. Analyst.

[44] Ma S., Ludwig R. (2019). Direct electron transfer of enzymes facilitated by cytochromes. ChemElectroChem.

[45] Rahman M.A., Park D.-S., Chang S.-C., McNeil C.J., Shim Y.-B. (2006). The biosensor based on the pyruvate oxidase modified conducting polymer for phosphate ions determinations. Biosens. Bioelectron..

[46] Suen H.-M.E., Pasvol G., Cunnington A.J. (2020). Clinical and laboratory features associated with serum phosphate concentrations in malaria and other febrile illnesses. Malar. J..

[47] Zou Z., Han J., Jang A., Bishop P.L., Ahn C.H. (2007). A disposable on-chip phosphate sensor with planar cobalt microelectrodes on polymer substrate. Biosens. Bioelectron..

[48] Cinti S., Talarico D., Palleschi G., Moscone D., Arduini F. (2016). Novel reagentless paper-based screen-printed electrochemical sensor to detect phosphate. Anal. Chim. Acta.

[49] Talarico D., Arduini F., Amine A., Moscone D., Palleschi G. (2015). Screen-printed electrode modified with carbon black nanoparticles for phosphate detection by measuring the electroactive phosphomolybdate complex. Talanta.

[50] Kwan R.C., Leung H.F., Hon P.Y., Barford J.P., Renneberg R. (2005). A screen-printed biosensor using pyruvate oxidase for rapid determination of phosphate in synthetic wastewater. Appl. Microbiol. Biotechnol..

[51] Fernández J.J., López J.R., Correig X., Katakis I. (1998). Reagentless carbon paste phosphate biosensors: Preliminary studies. Sens. Actuators B Chem..

[52] Upadhyay L.S.B., Verma N. (2015). Recent advances in phosphate biosensors. Biotechnol. Lett..

[53] Norouzi P., Pirali-Hamedani M., Faridbod F., Ganjali M.R. (2010). Flow Injection Phosphate Biosensor Based on PyOx-MWCNTs Film on a Glassy Carbon Electrode Using FFT Continuous Cyclic Voltammetry. Int. J. Electrochem. Sci..

[54] Gavalas V.G., Chaniotakis N.A. (2001). Phosphate biosensor based on polyelectrolyte-stabilized pyruvate oxidase. Anal. Chim. Acta.

[55] Wang Z., Liu Q., Zhu H., Liu H., Chen Y., Yang M.J.C. (2007). Dispersing multi-walled carbon nanotubes with water–soluble block copolymers and their use as supports for metal nanoparticles. Carbon.

[56] NB R.K., Crasta V., Praveen B., Kumar M.J.N.R. (2015). Studies on structural, optical and mechanical properties of MWCNTs and ZnO nanoparticles doped PVA nanocomposites. Nanotechnol. Rev..

[57] Vuković G.D., Marinković A.D., Čolić M., Ristić M.Đ., Aleksić R., Perić-Grujić A.A., Uskoković P.S. (2010). Removal of cadmium from aqueous solutions by oxidized and ethylenediamine-functionalized multi-walled carbon nanotubes. Chem. Eng. J..

