# Supplementary material for: Enzyme Immobilization by Inkjet Printing on Reagentless Biosensors for Electrochemical Phosphate Detection
Source: Biosensors (Basel). 2024 Mar 30;14(4):168. doi: 10.3390/bios14040168 (PMC11047959; doi:10.3390/bios14040168)
Supplement: Supplementary file 1 [file biosensors-14-00168-s001.zip › biosensors-2845313-supplementary.pdf]

Supporting information

Enzyme immobilization by inkjet printing on reagentless biosensors for electrochemical phosphate detection

Dongxing Zhang[a†], Yang Bai[b†], Haoran Niu[a], Lingyun Chen[a], Junfeng Xiao[b], Peipei Jia[a*], Qiuquan Guo[a*]

[a.] Shenzhen Institute for Advanced Study, University of Electronic Science and Technology of China. Yesun Industry Zone, Guanlan Street, Shenzhen, Guangdong, 518110, China.

[b.] Department of Biomedical Engineering, University of Western Ontario, 1151 Richmond Street, London, Ontario N6A 3K7, Canada.

* Corresponding author.

† These authors contributed equally to the work.

1. Characterization of the functionalized MWCNTs

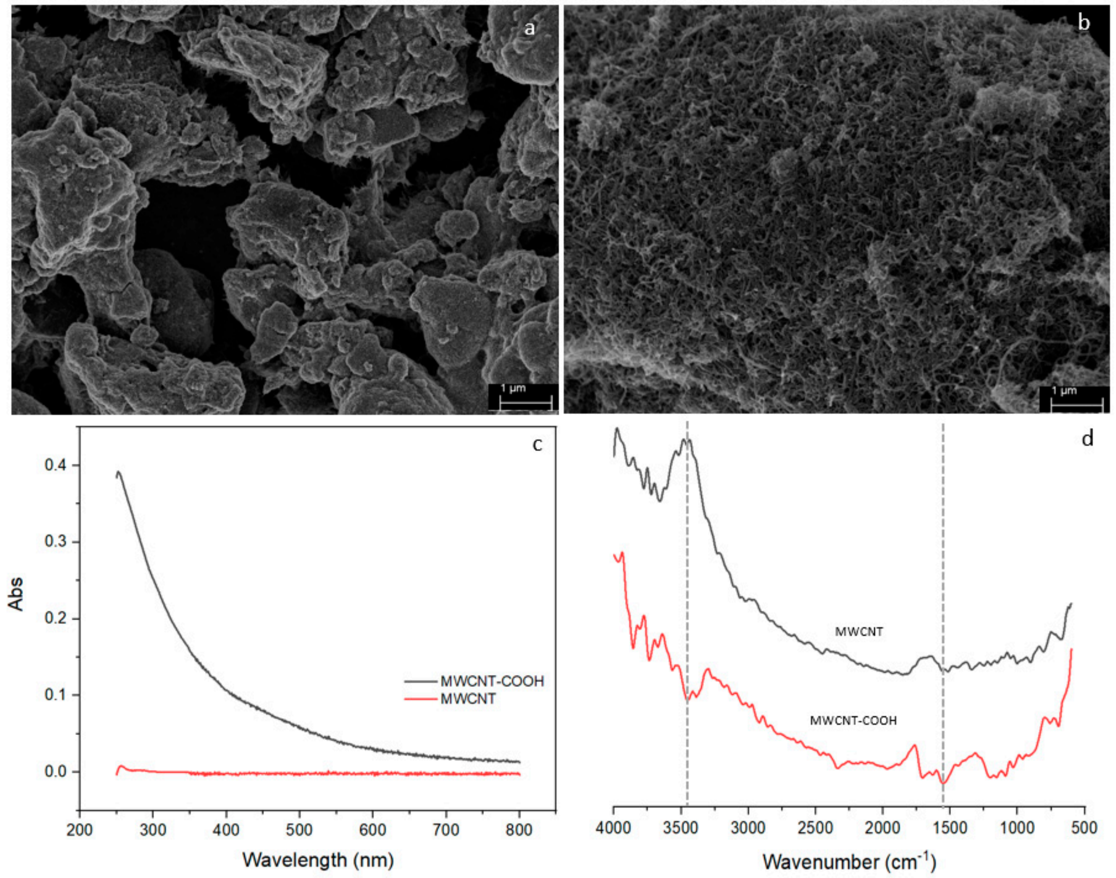

Figure S1 (a) SEM images of the aggregated MWCNTs. (b) SEM images of the MWCNTs-COOH. (c) UV-vis absorption spectra of the MWCNTs solution and the MWCNTs-COOH solution. (d) FTIR of the MWCNTs and the MWCNTs-COOH.

Compared with the non-modified MWCNTs, which aggerated easily on the substrate due to its hydrophobic property (Figure S1a), the functionalized MWCNTs have a better distribution (Figure S1b). UV-vis absorption spectrums were further utilized to compare the improvement, as shown in Figure S1c. Both CNT solutions (5 mg/ml) were filtered by 0.2 µm filter due to the requirement of ink printability. The absorbance of filtered non-modified MWCNT solution was almost same with the buffer solution (~ 0) meaning that most of the MWCNTs were filtered away due to severe aggregation. The absorbance of the filtered modified MWCNT solution showed a regular increasing tendency towards lower wavelength demonstrating the high dispersion property after functionalization [1]. The characteristic peaks at 3438 and 1610 cm^{-1} in the FTIR transmission spectrum of MWCNTs-COOH indicated that the stretching vibrations of O-H and C=O of carboxyl groups (Figure 3d) [2]. Occurrence of these peaks in the FTIR spectrum suggested the successful inclusion of -COOH groups on the external surface of MWCNTs [3].

2. Printing waveforms for different functional layers

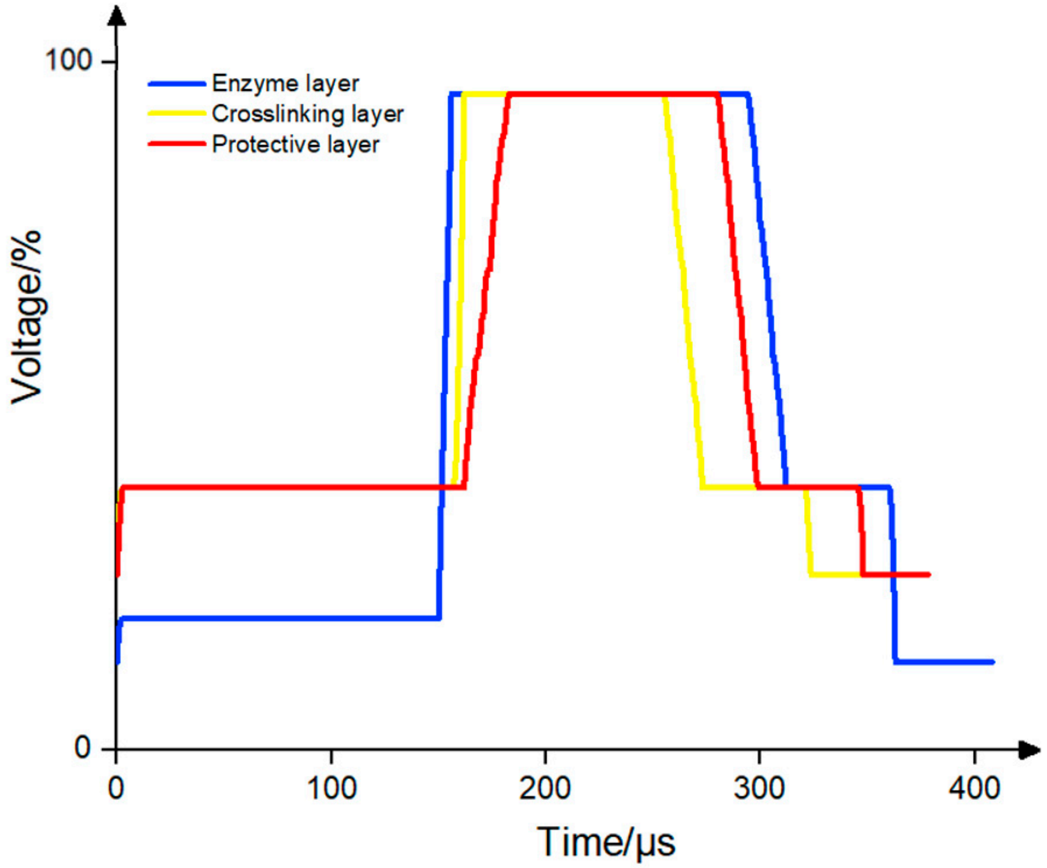

Figure S2 Printing waveforms for enzyme layer (blue line), crosslinking layer (yellow line), and protective layer (red line).

3. Cost breakdown for the prosed biosensor

Table S1 Cost breakdown of the serum phosphate sensor (materials and reagents)

Material	Stock volume/Amount	Price ($)	Estimated volume/Amount for each sensor	Estimated price for each sensor ($)
**Screen printed sensor**	Pack of 25	30.58	1	1.22
**Triton X-100**	500 ml	44.69	0.02 µl	< 0.01
**GLA**	500 ml	31.20	0.72 µl	< 0.01
**Pyruvate oxidase**	1 KU	300	0.10 U	< 0.01
**Puruvid acid**	500 ml	100.25	< 0.01 µl	< 0.01
**$MgCl_2$**	1 kg	40.87	< 0.01 mg	< 0.01
**FAD**	25 mg	50.78	< 0.001 mg	< 0.01
**TPP**	5 g	54.83	< 0.001 mg	< 0.01
**BSA**	10 g	59.51	0.19 mg	< 0.01
**MWCNT**	100 g	27.52	0.004 mg	< 0.01
**Citric acid**	500 g	44.85	0.04 mg	< 0.01
**Sodium Citrate dihydrate**	1000 g	54.60	< 0.01 mg	< 0.01
**Nafion**	25 ml	106.08	0.01 µl	< 0.01
				Total < 1.34

Reference

1. Wang, Z., Q. Liu, H. Zhu, H. Liu, Y. Chen, and M.J.C. Yang, *Dispersing multi-walled carbon nanotubes with water–soluble block copolymers and their use as supports for metal nanoparticles.* 2007. **45**(2): p. 285-292.
2. NB, R.K., V. Crasta, B. Praveen, and M.J.N.R. Kumar, *Studies on structural, optical and mechanical properties of MWCNTs and ZnO nanoparticles doped PVA nanocomposites.* 2015. **4**(5): p. 457-467.
3. Vuković, G.D., A.D. Marinković, M. Čolić, M.Đ. Ristić, R. Aleksić, A.A. Perić-Grujić, and P.S. Uskoković, *Removal of cadmium from aqueous solutions by oxidized and ethylenediamine-functionalized multi-walled carbon nanotubes.* Chemical Engineering Journal, 2010. **157**(1): p. 238-248.
